# Correction to “Binary Mixtures of *n*‑Alkylbenzenes and Pentadecane: Densities, Speeds of Sound, and Viscosities within the Range of 288.15 and 333.15 K and at 0.1 MPa”

**DOI:** 10.1021/acs.jced.5c00652

**Published:** 2025-11-20

**Authors:** Dianne J. Luning Prak, Jim S. Cowart

Table 2: The correct CAS number for *n-*decylbenzene (C_16_H_26_) is 104-72-3.

**Table 2:** Chemical Information

chemical name	CAS number	molar mass (g/mol)^a^	source	mole fraction purity^b^
pentadecane (C_15_H_32_)	629-62-9	212.41 ± 0.04	TCI	0.998
*n-*decylbenzene (C_16_H_26_)	104-72-3	218.38 ± 0.03	TCI	0.994
*n-*octylbenzene (C_14_H_22_)	2189-60-8	190.32 ± 0.03	TCI	0.982
*n-*heptylbenzene (C_13_H_20_)	1078-71-3	176.30 ± 0.03	TCI	0.995
*n-*hexylbenzene (C_12_H_18_)	1077-16-3	162.27 ± 0.02	TCI	0.993
*n-*pentylbenzene (C_11_H_16_)	538-68-1	148.25 ± 0.02	Aldrich	0.999
*n-*butylbenzene (C_10_H_14_)	104-51-8	134.22 ± 0.02	TCI	0.999
*n-*propylbenzene (C_9_H_12_)	103-65-1	120.20 ± 0.02	TCI	0.999
ethylbenzene (C_8_H_10_)	100-41-4	106.17 ± 0.02	TCI	1.000
toluene (C_7_H_8_)	108-88-3	92.14 ± 0.01	Pharmco	0.9987

a Calculated using values in Harris.^30^

b The method of analysis for all the compounds is gas–liquid chromatography, as specified in the Certificates of Analysis provided by the chemical suppliers.

Table 5: The units and symbol for speed of sound in the table were incorrect. They should be m·s^–1^ and *c*.

**Table 5:** Measured Speeds of Sound *c* of *n*-Alkylcyclohexanes and *n*-Alkylbenzenes Compared with Literature Values at Temperature *T* and Pressure *p* = 0.1 MPa[Table-fn t5fn2]

*T*/K	Meas.[Table-fn t5fn2] *c*/m·s^–1^	Literature *c*/m·s^–1^	Meas.[Table-fn t5fn2] *c*/m·s^–1^	Literature *c*/m·s^–1^
	pentadecane		*n*-octylbenzene	
288.15	1364.3	1364.0,^34^ 1364.2^16^	1416.7	1415.9^36^
293.15	1345.3	1344.9,^34^ 1345.2,^16^ 1345.9^37^	1398.4	1397.3,^36^ 1397.5^8^
298.15	1326.3	1325.8,^34^ 1326.5^16^	1379.8	1378.7^36^
303.15	1307.5	1307.0,^34^ 1307.3,^53^ 1307.5,^16^ 1308.1^37^	1361.5	1360.2,^36^ 1361.0^8^
313.15	1270.8	1269.9,^53^ 1270.1,^34^ 1270.7,^16^, 1270.9^37^	1325.5	1324.1,^36^ 1324.9^8^
323.15	1234.7	1234.0,^34^ 1234.1,^53^ 1234.4,^16^ 1234.5^37^	1290.1	1288.6,^36^ 1289.4^8^
333.15	1199.2	1197.6^53^, 1198.3^37^, 1198.9^34^, 1199.1^16^	1255.1	1254.2,^36^ 1254.3^8^
		MAPE = 0.03		MAPE = 0.07
	*n*-heptylbenzene		*n*- hexylbenzene	
288.15	1405.8	1405.8^36^	1394.9	1395.0,^36^ 1395.8,^33^ 1395.6^33^
293.15	1387.1	1387.0^36^	1376.1	1375.9,^36^ 1376.0,^33^ 1376.1,^8^ 1376.5^33^
298.15	1368.4	1368.2^36^	1357.0	1356.9^36^
303.15	1349.9	1349.6^36^	1338.3	1338.0,^36^ 1338.1,^33^ 1338.7^8,33^
313.15	1313.5	1313.0^36^	1301.4	1301.0,^36^ 1301.1,^33^ 1301.6,^33^ 1301.7^8^
323.15	1277.7	1277.2^36^	1265.1	1264.7,^33^ 1265.3^8,33^
333.15	1242.4	1242.1^36^	1229.2	1229.3,^36^ 1229.4,^8,33^ 1229.8^33^
		MAPE = 0.02		MAPE = 0.02
	*n*-pentylbenzene		*n*-butylbenzene	
288.15	1385.4	1385.2^36^	1373.8	1373.7^33^
293.15	1366.0	1365.8^36^	1354.0	1353.2,^33^ 1353.4,^54^ 1353.8^55^
298.15	1346.7	1346.4,^36^ 1346^46^	1334.3	1334.1^55^
303.15	1327.5	1327.1^36^	1314.8	1308,^48^ 1314.3,^33,54^ 1314.5^55^
313.15	1289.9	1289.4^36^	1276.5	1275.7,^54^ 1276,^48^ 1276.1^33^
323.15	1252.8	1252.3^36^	1238.7	1238.4^33^
333.15	1216.2	1216.2^36^	1201.5	1201.5,^33^ 1201^55^
		MAPE = 0.03		MAPE = 0.05
	*n*-propylbenzene		ethylbenzene	
288.15	1362.0	1361.3^36, 49^	1361.3	1361.0^36^
293.15	1341.6	1340.7,^49^ 1340.8,^36^ 1341.5^14^	1340.2	1339.70,^26^ 1339.9,^36^ 1340^56^
298.15	1321.2	1320.6,^14^ 1320.3,^36^ 1321.0^57^	1319.1	1318.8^36^
303.15	1301.1	1300.0,^49^ 1300.1,^36^ 1300.4^14^	1298.2	1298.0,^36^ 1298.05^26^
313.15	1261.5	1260.2,^36,49^ 1260.9^14^	1257.1	1256,^58^ 1256.89,^26^ 1257,^56^ 1257.2^36^
323.15	1222.4	1221.2,^36,49^ 1221.5^14^	1216.7	1216.35^26^
333.15	1183.9	1183.1^36,49^	1176.8	1176.40^26^
		MAPE = 0.07		MAPE = 0.03
	toluene			
288.15	1348.3	1348.2^36^		
293.15	1326.6	1326.5,^36^ 1326.8,^26^ 1326.9^59^		
298.15	1304.8	1304.7,^36^ 1304.77 ± 0 × 10^60^		
303.15	1283.2	1283,^61^ 1283.20 ± 0 × 10^60^		
		MAPE = 0.01		

a “Meas.” is measured value. The average pressure for these measurements was 0.102 MPa. Standard uncertainties are *u*(*T*) = 0.01 K, and *u*(*p*) = 1 kPa, and relative standard uncertainties are *u*_*r*_(*c*) = 0.0007, except for *n*-octylbenzene mixtures where the relative standard uncertainties *u*_*r*_(*c*) = 0.0018.

Table 8: The units in the header were incorrect. They should be m·s^–1^.

**Table 8:** Speeds of Sound *c* (m·s^–1^) of Binary Mixtures of an *n*-Alkylbenzene at Mole Fraction *x*_*1*_ in Pentadecane at Temperature *T* and Pressure of 0.1 MPa^a^

*n*-alkylbenzene	*x*_1_	*T* = 288.15 K	*T* = 293.15 K	*T* = 298.15 K	*T* = 303.15 K	*T* = 313.15 K	*T* = 323.15 K	*T* = 333.15 K
	0.0000	1364.3	1345.3	1326.3	1307.5	1270.8	1234.7	1199.2
toluene	0.1017	1359.8	1340.9	1321.8	1303.0			
toluene	0.1987	1355.6	1336.5	1317.5	1298.6			
toluene	0.2985	1351.1	1332.0	1312.9	1293.9			
toluene	0.3992	1346.5	1327.3	1308.1	1289.1			
toluene	0.5014	1342.1	1322.7	1303.3	1284.1			
toluene	0.5996	1338.1	1318.6	1299.0	1279.6			
toluene	0.7000	1334.9	1315.2	1295.4	1275.7			
toluene	0.8004	1333.9	1313.8	1293.5	1273.5			
toluene	0.9010	1336.9	1316.1	1295.3	1274.6			
toluene	1.0000	1348.3	1326.6	1304.8	1283.2			
ethyl-	0.1000	1360.6	1341.5	1322.6	1303.8	1267.0	1230.9	1195.2
ethyl-	0.2010	1357.0	1338.0	1319.0	1300.2	1263.3	1227.1	1191.4
ethyl-	0.3002	1353.6	1334.6	1315.5	1296.7	1259.6	1223.3	1187.4
ethyl-	0.3994	1350.5	1331.4	1312.2	1293.3	1256.1	1219.5	1183.5
ethyl-	0.5011	1347.5	1328.3	1309.1	1290.2	1252.8	1215.9	1179.6
ethyl-	0.6001	1345.6	1326.2	1306.8	1287.6	1249.9	1212.7	1176.0
ethyl-	0.7002	1344.6	1325.0	1305.4	1286.1	1247.9	1210.3	1173.1
ethyl-	0.7990	1345.9	1325.9	1306.0	1286.2	1247.4	1209.1	1171.4
ethyl-	0.9003	1350.2	1329.9	1309.5	1289.4	1249.8	1210.6	1172.0
ethyl-	1.000	1361.3	1340.2	1319.1	1298.2	1257.1	1216.7	1176.8
propyl-	0.1020	1361.1	1342.1	1323.1	1304.4	1267.6	1231.5	1195.9
propyl-	0.2021	1358.3	1339.3	1320.3	1301.6	1264.7	1228.5	1192.8
propyl-	0.3075	1355.4	1336.4	1317.5	1298.7	1261.8	1225.5	1189.7
propyl-	0.4001	1353.2	1334.2	1315.2	1296.4	1259.4	1223.0	1187.1
propyl-	0.4998	1351.2	1332.1	1313.0	1294.2	1257.0	1220.4	1184.3
propyl-	0.6006	1349.9	1330.7	1311.5	1292.5	1255.1	1218.2	1181.9
propyl-	0.6985	1349.5	1330.2	1310.8	1291.7	1254.0	1216.9	1180.2
propyl-	0.8001	1350.7	1331.1	1311.5	1292.2	1254.1	1216.5	1179.5
propyl-	0.8997	1354.4	1334.4	1314.5	1294.8	1256.0	1217.9	1180.3
propyl-	1.0000	1362.0	1341.6	1321.2	1301.1	1261.5	1222.4	1183.9
butyl-	0.1004	1362.5	1343.4	1324.4	1305.7	1268.9	1232.9	1197.3
butyl-	0.2175	1360.4	1341.6	1322.5	1303.9	1267.3	1231.2	1195.6
butyl-	0.3011	1359.3	1340.5	1321.5	1302.9	1266.2	1230.0	1194.4
butyl-	0.4011	1358.5	1339.5	1320.6	1301.9	1265.1	1228.9	1193.3
butyl-	0.5065	1357.9	1339.0	1320.1	1301.4	1264.6	1228.3	1192.5
butyl-	0.5999	1358.3	1339.4	1320.3	1301.6	1264.6	1228.2	1192.2
butyl-	0.7004	1359.5	1340.5	1321.4	1302.6	1265.4	1228.8	1192.6
butyl-	0.7994	1362.2	1342.9	1323.7	1304.6	1267.1	1230.3	1193.9
butyl-	0.8994	1366.4	1347.2	1327.6	1308.5	1270.7	1233.5	1196.8
butyl-	1.0000	1373.8	1354.0	1334.3	1314.8	1276.5	1238.7	1201.5
pentyl-	0.4999	1364.8	1345.8	1326.9	1308.2	1271.3	1235.2	1199.6
pentyl-	1.0000	1385.4	1366.0	1346.7	1327.5	1289.9	1252.8	1216.2
hexyl-	0.1001	1364.7	1345.7	1326.8	1308.1	1271.3	1235.3	1199.8
hexyl-	0.2002	1365.4	1346.5	1327.6	1309.0	1272.4	1236.4	1200.9
hexyl-	0.3007	1366.6	1347.8	1328.9	1310.4	1273.9	1237.9	1202.5
hexyl-	0.4011	1368.4	1349.5	1330.7	1312.1	1275.6	1239.7	1204.3
hexyl-	0.5002	1370.6	1351.8	1333.0	1314.4	1277.9	1241.9	1206.5
hexyl-	0.5997	1373.4	1354.7	1335.8	1317.3	1280.8	1244.8	1209.4
hexyl-	0.6996	1377.2	1358.3	1339.5	1320.9	1284.3	1248.3	1212.8
hexyl-	0.8003	1381.8	1363.0	1344.1	1325.5	1288.9	1252.8	1217.2
hexyl-	0.8996	1387.6	1368.8	1349.8	1331.1	1294.4	1258.2	1222.5
hexyl-	1.0000	1394.9	1376.1	1357.0	1338.3	1301.4	1265.1	1229.2
heptyl-	0.4996	1376.9	1358.3	1339.5	1321.0	1284.7	1249.0	1213.7
heptyl-	1.0000	1405.8	1387.1	1368.4	1349.9	1313.5	1277.7	1242.4
octyl-	0.0000	1364.3	1345.3	1326.3	1307.5	1270.8	1234.7	1199.2
octyl-	0.1013	1367.2	1348.5	1329.5	1310.9	1274.3	1238.3	1202.8
octyl-	0.2002	1370.7	1351.9	1333.0	1314.4	1277.9	1242.0	1206.7
octyl-	0.3589	1376.9	1358.3	1339.5	1321.0	1284.7	1249.0	1213.7
octyl-	0.4554	1381.2	1362.8	1344.1	1325.6	1289.4	1253.6	1218.5
octyl-	0.5010	1383.7	1364.9	1346.2	1327.8	1291.6	1256.0	1220.8
octyl-	0.5999	1388.8	1370.2	1351.6	1333.2	1297.1	1261.5	1226.4
octyl-	0.7000	1394.6	1376.3	1357.7	1339.3	1303.2	1267.7	1232.7
octyl-	0.7998	1401.3	1382.7	1364.1	1345.8	1309.8	1274.3	1239.3
octyl-	0.8990	1408.6	1390.0	1371.4	1353.1	1317.1	1281.7	1246.7
octyl-	1.0000	1416.7	1398.4	1379.8	1361.5	1325.5	1290.1	1255.1
decyl-	0.4987	1394.5	1375.9	1357.4	1339.1	1303.0	1267.7	1232.9
decyl-	1.0000	1433.5	1415.2	1396.9	1378.9	1343.5	1308.7	1274.4

a *x*_1_ is the mole fraction of an *n*-alkylbenzene (1) in pentadecane (2). The average pressure for these measurements was 0.102 MPa. Standard uncertainties are *u*(*T*) = 0.01 K and *u*(*p*) = 1 kPa, relative standard uncertainties are *u*_*r*_(*c*) = 0.0018 for *n*-octylbenzene and *u*_*r*_(*c*) = 0.0007 for all other mixtures and combined expanded uncertainty *U*(*x*_1_) = 0.0001 (level of confidence = 0.95, *k* = 2).

